# The role of Lrp6-mediated Wnt/β-catenin signaling in the development and intervention of spinal neural tube defects in mice

**DOI:** 10.1242/dmm.049517

**Published:** 2022-06-10

**Authors:** Tianyu Zhao, Moira McMahon, Kurt Reynolds, Subbroto Kumar Saha, Arjun Stokes, Chengji J. Zhou

**Affiliations:** Institute for Pediatric Regenerative Medicine of Shriners Hospitals for Children-Northern California, Department of Biochemistry and Molecular Medicine, University of California, Davis School of Medicine, Sacramento, CA 95817, USA

**Keywords:** Lrp6, Wnt/β-catenin signaling, Spinal neural tube defects, Genetic rescue, Pharmacological intervention

## Abstract

Neural tube defects (NTDs) are among the common and severe birth defects with poorly understood etiology. Mutations in the Wnt co-receptor *LRP6* are associated with NTDs in humans. Either gain-of-function (GOF) or loss-of-function (LOF) mutations of *Lrp6* can cause NTDs in mice. NTDs in *Lrp6*-GOF mutants may be attributed to altered β-catenin-independent noncanonical Wnt signaling. However, the mechanisms underlying NTDs in *Lrp6*-LOF mutants and the role of Lrp6-mediated canonical Wnt/β-catenin signaling in neural tube closure remain unresolved. We previously demonstrated that β-catenin signaling is required for posterior neuropore (PNP) closure. In the current study, conditional ablation of *Lrp6* in dorsal PNP caused spinal NTDs with diminished activities of Wnt/β-catenin signaling and its downstream target gene *Pax3*, which is required for PNP closure. β-catenin-GOF rescued NTDs in *Lrp6*-LOF mutants. Moreover, maternal supplementation of a Wnt/β-catenin signaling agonist reduced the frequency and severity of spinal NTDs in *Lrp6*-LOF mutants by restoring *Pax3* expression. Together, these results demonstrate the essential role of Lrp6-mediated Wnt/β-catenin signaling in PNP closure, which could also provide a therapeutic target for NTD intervention through manipulation of canonical Wnt/β-catenin signaling activities.

## Abstract (precis)

**Summary:** Conditional ablation of *Lrp6* in dorsal neural folds causes spinal neural tube defects that can be rescued by genetic activation of β-catenin or maternal supplementation of Wnt signaling agonists.

## INTRODUCTION

The neural tube is the precursor structure of the brain and spinal cord. Failure of proper neural tube closure at different anatomical regions along the anterior–posterior body axis results in various types of severe neural tube defects (NTDs), such as spina bifida (spinal NTDs) and exencephaly (cranial NTDs), the former of which is the most common type of NTD in humans (Greene and Copp, 2014). Although NTDs affect more than 300,000 newborns worldwide annually, of which ∼3000 cases occur in the United States (Centers for Disease and Prevention (CDC), 2004; Zaganjor et al., 2016), the etiology of NTDs remains poorly understood due to its complexity (Copp et al., 2013; Wallingford et al., 2013). Folate supplementation may prevent a considerable portion of NTDs, but the majority of NTDs that occurred in developed countries with mandatory folic acid fortification are considered unpreventable by folate intake alone (Williams et al., 2015). Therefore, it is imperative to study fundamental mechanisms of normal and defective neural tube closure, which could lead to the development of novel prevention strategies for NTDs.

Mutant mice have been widely used for NTD mechanistic and preventative studies (Greene and Copp, 2005; Zohn, 2020). More than 200 genes have been linked with NTDs in mice (Harris and Juriloff, 2007, 2010), suggesting that the genetic basis of neural tube closure is highly complex. However, only a small number of genes, including several involved in Wnt signaling, have been associated with human NTDs (Allache et al., 2014; Au et al., 2010; De Marco et al., 2014).

Morphogenetic Wnt signaling plays vital roles in early embryonic development, including gastrulation and neurulation. Wnt ligands bind to various types of receptors and co-receptors to transduce signals via β-catenin-dependent (canonical) and β-catenin-independent (noncanonical) pathways. The noncanonical Wnt/planar cell polarity (PCP) signaling pathway regulates cytoskeleton dynamics and collective tissue movements, such as convergent extension, which may drive neural tube closure (Copp et al., 2003; Wallingford, 2006; Wang et al., 2019; Ybot-Gonzalez et al., 2007). The rare but severest type of NTDs, craniorachischisis, is characterized by an entirely open brain and spinal cord (Tobin et al., 2019), and results from failure of the initial neural tube closure at the boundary between the future hindbrain and spinal cord. Craniorachischisis is associated with defective convergent extension and has been mainly found in mice with mutant PCP signaling genes, such as *Ptk7* and the Vangl, Celsr, Dvl and Fzd families (De Marco et al., 2011; Juriloff and Harris, 2012). Several of these PCP components, such as Fzds and Dvls, also play essential roles in the canonical Wnt/β-catenin signaling pathway (MacDonald et al., 2009).

The Wnt co-receptor Lrp6 acts upstream of β-catenin in the canonical pathway and is required for a wide range of processes pertaining to embryogenesis and organogenesis, including neural tube closure (Alrefaei and Abu-Elmagd, 2022; Carter et al., 2005; He et al., 2004; Kokubu et al., 2004; Mao et al., 2001; Pinson et al., 2000; Song et al., 2009, 2010; Tamai et al., 2000; Wang et al., 2016; Wehrli et al., 2000; Zhou et al., 2008, 2004, 2010). Multiple studies have identified mutations in human *LRP6* gene from patients with NTDs (Allache et al., 2014; Lei et al., 2015; Shi et al., 2018). In mice, both hypermorphic and hypomorphic mutations in *Lrp6* gene can cause NTDs. The cranial NTDs in the spontaneous mutant *crooked tail* mice (Carter et al., 2005) and spinal NTDs in novel N-ethyl-N-nitrosourea (ENU)-induced *Skax26* mice (in combination with heterozygous *Vangl2^Lp^*) (Allache et al., 2014) are caused by hypermorphic *Lrp6*. In contrast, spinal NTDs in the spontaneous *ringelschwanz* mice are caused by hypomorphic *Lrp6* (Kokubu et al., 2004), and *Lrp6*-null mutants exhibit partially penetrant cranial NTDs and fully penetrant spinal NTDs (Pinson et al., 2000; Zhou et al., 2010). It has been reported that altered noncanonical Wnt signaling may contribute to NTDs in hypermorphic *Lrp6* mutants (Allache et al., 2014; Gray et al., 2013). However, the causal mechanisms of NTDs in *Lrp6*-deficient mutants and the explicit role of Lrp6-mediated canonical Wnt/β-catenin signaling in neural tube closure are thus far obscure.

We have previously demonstrated that conditional ablation of β-catenin in the dorsal neural folds diminishes expression of the paired-box gene *Pax3*, which encodes a transcription factor critical to neural tube closure, resulting in an open spinal NTD phenotype (Zhao et al., 2014). The β-catenin-Tcf/Lef1 complex modulates activity of the *Pax3* promoter, and ectopic activation of *Pax3* transgene can rescue spinal NTDs in β-catenin mutants. Moreover, β-catenin and Pax3 cooperate to regulate the caudal type of homeobox gene *Cdx2* for caudal body axis elongation (Zhao et al., 2014). In the current study, we demonstrate that conditional ablation of *Lrp6* in dorsal neural folds causes fully penetrant spinal NTDs, as a potential consequence of diminished activities of canonical Wnt/β-catenin signaling and its downstream target gene *Pax3*, which are consistent with those occurring in conditional β-catenin mutants. Moreover, our genetic and pharmacological rescue approaches demonstrate the crucial roles of Lrp6-mediated Wnt/β-catenin signaling in the development of spinal NTDs.

## RESULTS

### Conditional gene targeting of *Lrp6* in Pax3-expressing dorsal neural fold causes posterior neuropore (PNP) closure defects and diminished canonical Wnt/β-catenin signaling

**Fig. 1.**
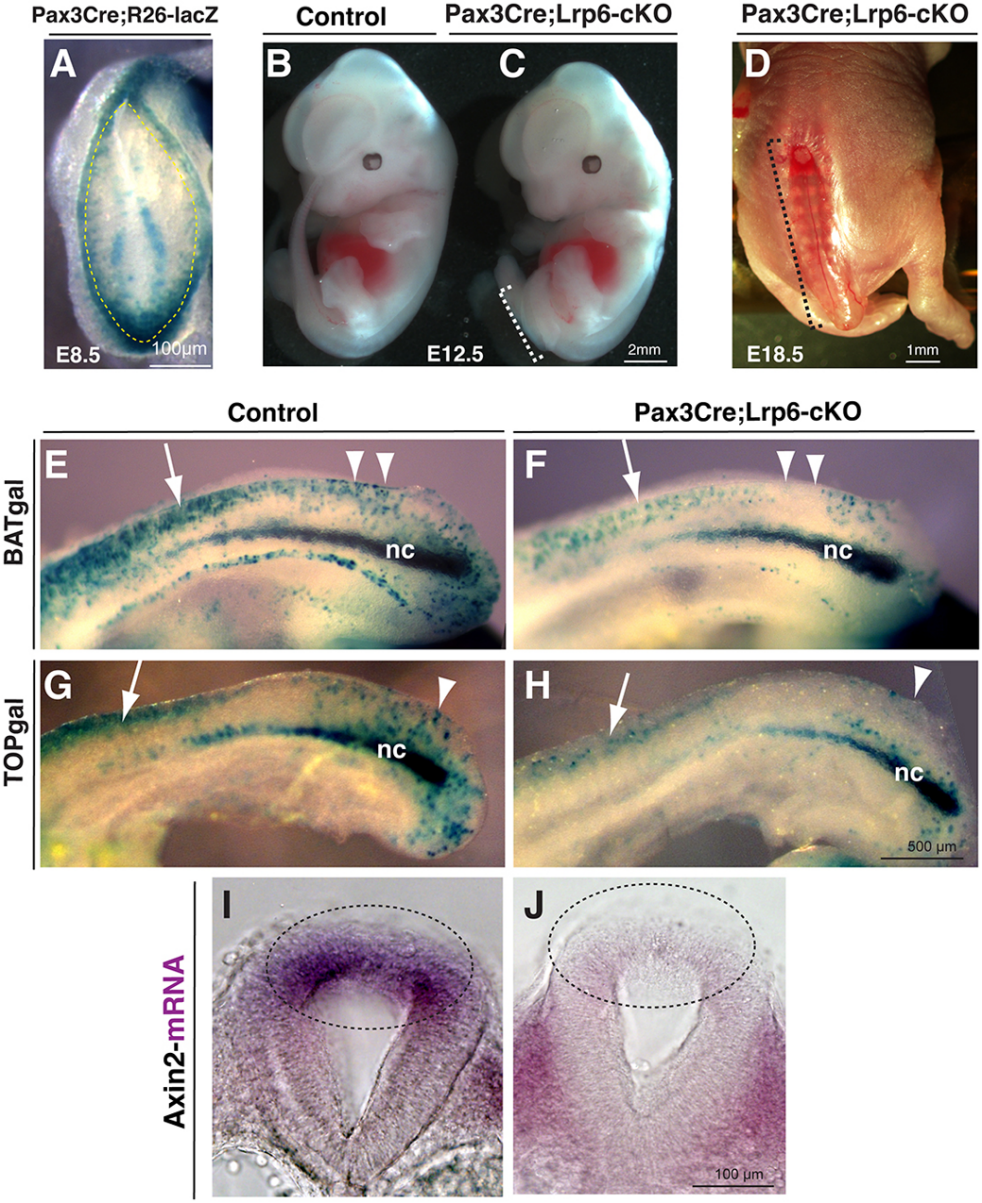
**Spinal bifida aperta and diminished canonical Wnt signaling by conditional ablation of *Lrp6* in Pax3-expressing dorsal neural folds.** (A) Dorsal–posterior view of an X-gal-stained (blue) E8.5 embryo for genetic fate mapping of *Pax3^Cre/+^;Rosa26-lacZ* demonstrates the Cre recombination pattern in the dorsal region of the recently closed and pending-closing posterior neuropore (PNP; indicated by dashed line). (B-D) The conditional mutants of *Pax3^Cre/+^;Lrp6*-cKO embryos exhibit open spinal neural tube defects (NTDs), as shown at E12.5 and E18.5. Dashed line brackets indicate the open lesion regions. (E-H) Sagittal caudal bodies of X-gal-stained Wnt/β-catenin signaling reporters *BATgal* or *TOPgal* show higher activities in the littermate control embryos (E,G) and diminished activities in the *Pax3^Cre/+^;Lrp6*-cKO embryos (F,H) at E9.5. Arrows indicate recently closed dorsal neural tube regions. Arrowheads indicate the closing or pending-closing regions. nc, notochord. (I,J) Transverse sections show *in situ* hybridization signal of a Wnt/β-catenin target and feedback gene *Axin2*, which is high in the dorsal PNP of a littermate control (dashed line oval in I) and low in the mutant PNP (dashed line oval in J) at E9.5.

To determine the cell lineage-specific role of Lrp6 in neural tube closure, we carried out conditional gene-targeting analyses by breeding *Lrp6^flox^* mice (Zhou et al., 2010) with the *Pax3^Cre^* knock-in mice (Lang et al., 2005). Genetic fate mapping of *Pax3^Cre^* mice crossed with the Cre reporter *Rosa26-lacZ* revealed restrictive activities of the Cre recombinase in the dorsal neural folds, including in the recently closed dorsal midline region and the dorsal edge cells along the pending closure sites of the PNP of embryonic day (E)8.5 mouse embryos (Fig. 1A). Genetic fate mapping of *Pax3^Cre^* at E9.5 PNP has been detailed in our previous study (Zhao et al., 2014). The neural tube closes completely from E10.5 onwards in the non-Cre homozygous *Lrp6^flox/flox^* or in the double-heterozygous *Pax3^Cre/+^;Lrp6^flox/+^* littermate control mice (Fig. 1B). However, the conditional knockout *Pax3^Cre/+^;Lrp6^flox/flox^* (abbreviated as *Pax3Cre;Lrp6*-cKO or *Lrp6*-cKO) mice exhibit fully penetrant tail truncations and spinal bifida, as shown by the consistently open PNP at E12.5 and open caudal spinal cord at E18.5 (Fig. 1C,D). These results indicate that Lrp6 is required in the Pax3-expressing dorsal edge cells for PNP closure, which is consistent with the role of β-catenin in the same lineage for PNP closure, as we previously demonstrated (Zhao et al., 2014).

To validate whether Lrp6-mediated Wnt/β-catenin signaling is disrupted in the mutant PNP, we incorporated two representative Wnt/β-catenin signaling reporter mouse lines, *BATgal* (Maretto et al., 2003) and *TOPgal* (DasGupta and Fuchs, 1999), to the conditional gene-targeting approaches. X-gal staining for the signaling reporter lacZ of either *BATgal* or *TOPgal* indicated a clear reduction of Wnt/β-catenin signaling activities in the dorsal PNP of *Lrp6*-cKO embryos at E9.5, compared to that of their triple heterozygous littermate controls (Fig. 1E-H). The signaling reporter shows intensive Wnt/β-catenin signaling activities in the notochord (Ukita et al., 2009), which was not altered in the mutants as *Pax3^Cre^* is not expressed in the notochord. Because these signaling reporters may not visualize some less robust but functional Wnt/β-catenin signaling *in vivo*, we further examined the expression of *Axin2*, a general downstream target gene and negative feedback regulator of the canonical Wnt/β-catenin signaling pathway (Jho et al., 2002). Wholemount *in situ* hybridization showed a substantial reduction in *Axin2* mRNA at E9.5 in the dorsal PNP of *Lrp6*-cKO embryos compared to that in the littermate control embryos (Fig. 1I,J). These results demonstrate diminished Wnt/β-catenin signaling in *Lrp6*-deficient dorsal neural folds.

### NTD-associated transcription factors and β-catenin downstream target genes are diminished in the dorsal PNPs of *Pax3Cre;Lrp6*-cKO mutants

**Fig. 2.**
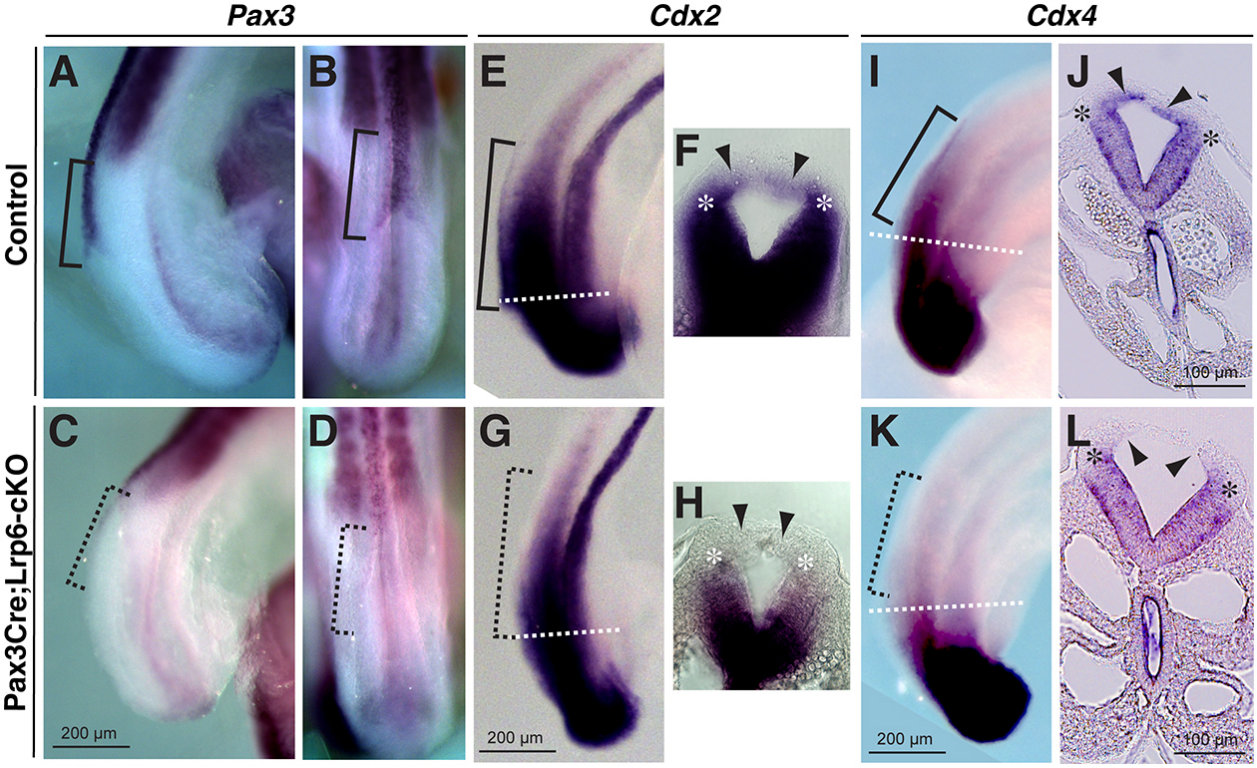
**Wholemount *in situ* hybridization results show diminished gene expression of NTD-associated transcription factors *Pax3*, *Cdx2* and *Cdx4* in the dorsal PNPs of *Pax3-Cre;Lrp6*-cKOs at E9.5.** (A-D) *Pax3* expression is strong at the PNP closure site, as shown in a littermate control embryo (brackets in A, sagittal view and in B, dorsal view), whereas it is diminished specifically at the defective closure site of the mutant PNP (dashed line brackets in C,D). (E-H) *Cdx2* is widely expressed in the caudal body of the control embryo, including dorsal PNP (bracket in E, sagittal view; arrowheads in F, transverse section from the region of the dashed line in E), and it is specifically diminished in the dorsal PNP of the mutant embryo (dashed line bracket in G and arrowheads in H). (I-L) *Cdx4* is expressed in the dorsal PNP of the control embryo (bracket in I and arrowheads in J), and it is specifically diminished in the dorsal PNP of the mutant embryo (dashed line bracket in K and arrowheads in L). Asterisks indicate the dorsolateral hinge points.

**Fig. 3.**
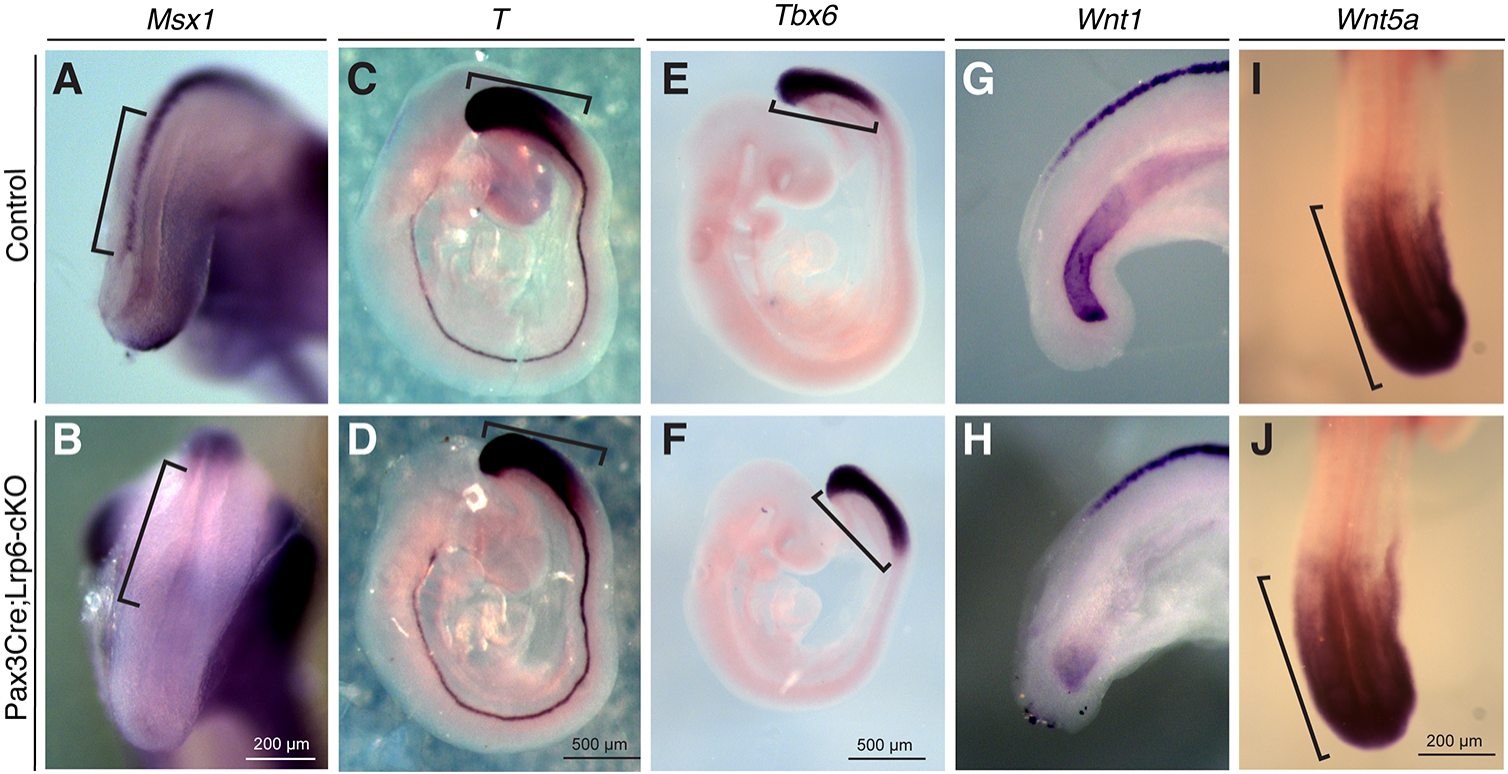
**Wholemount *in situ* hybridization results on Wnt genes and additionally relevant Wnt signaling downstream target genes around the PNP regions of littermate controls and *Pax3-Cre;Lrp6*-cKOs at E9.5.** (A,B) *Msx1* is restrictively expressed in the dorsal PNP of the normal control embryo (bracket in A), and its expression is significantly diminished in the mutant PNP (bracket in B). (C-J) No obvious changes in *T* (C,D), *Tbx6* (E,F), *Wnt1* (G,H) and *Wnt5a* (I,J) expression patterns around the mutant PNP regions were observed compared to respective expression patterns in the littermate controls.

To address the molecular mechanisms underlying spinal NTDs in *Lrp6*-cKO mutants, we examined a panel of NTD-associated genes that are specifically expressed in the dorsal neural folds, including the transcription factor *Pax3* (Epstein et al., 1991; Goulding et al., 1991) and the caudal-type homeobox genes *Cdx2* and *Cdx4* (Young et al., 2009; van Nes et al., 2006). Wholemount *in situ* hybridization showed diminished expression of *Pax3*, *Cdx2* and *Cdx4* in the dorsal neural folds of *Lrp6*-cKO embryos at E9.5 (Fig. 2). We have previously demonstrated that both *Pax3* and *Cdx2* are downstream target genes of β-catenin signaling, and *Cdx2* is additionally regulated by Pax3 during PNP closure (Zhao et al., 2014). The transcription factor Msx1 is a regulator of *Pax3* (Monsoro-Burq et al., 2005), and it is also a known β-catenin downstream effector (Foerst-Potts and Sadler, 1997; Song et al., 2009). *Msx1* was restrictively expressed in the dorsal midline during PNP closure, but it was absent in *Lrp6*-cKO PNPs at E9.5 (Fig. 3A,B). These results demonstrate the consistent roles of Lrp6 and β-catenin in regulation of these key downstream target genes during PNP closure.

**Fig. 4.**
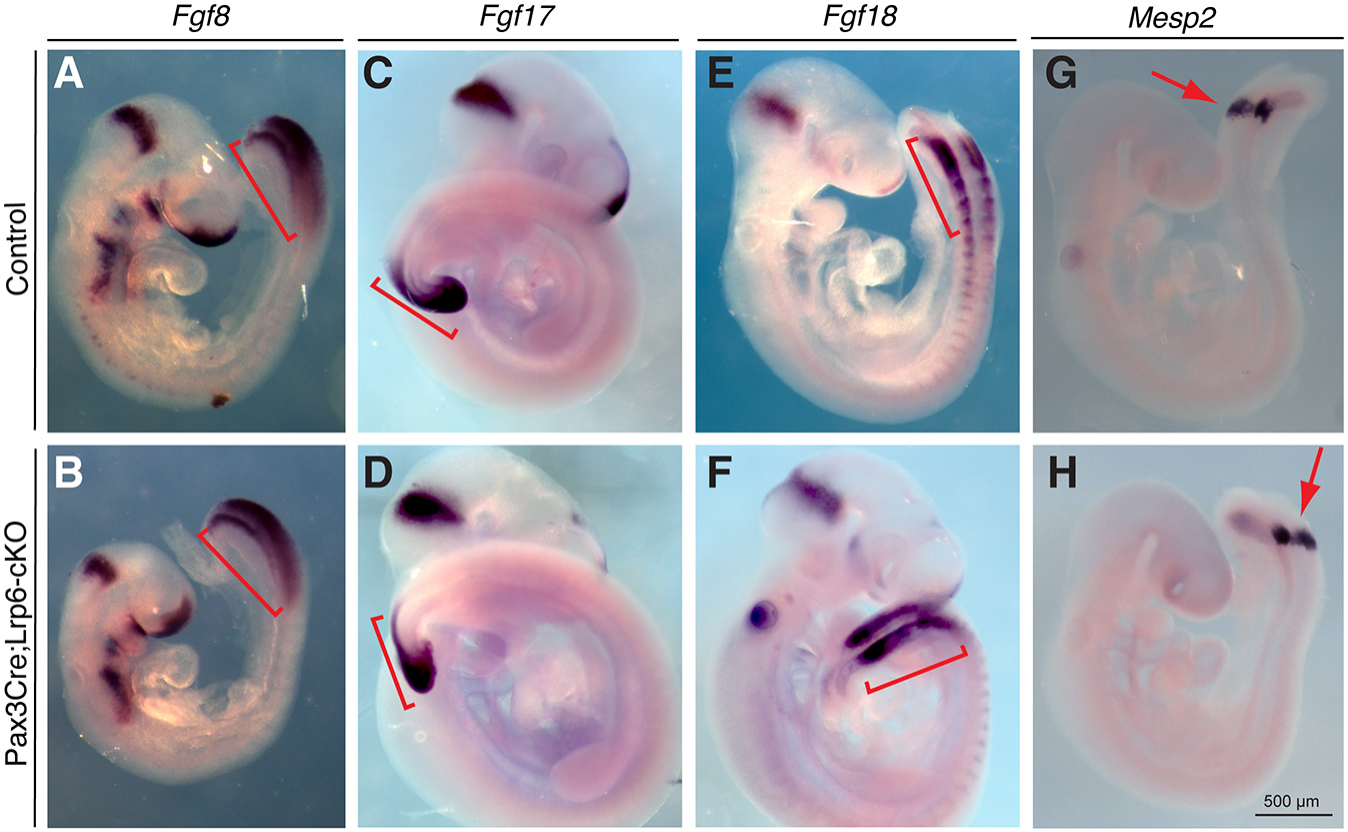
**Wholemount *in situ* hybridization results on Fgf genes and related *Mesp2* expression around PNP regions of the littermate controls and *Pax3-Cre;Lrp6*-cKOs at E9.5.** (A-F) No obvious differences in *Fgf8* (A,B), *Fgf17* (C,D) and *Fgf18* (E,F) expression around PNP regions (brackets) between the control and mutant embryos were observed. (G,H) No obvious differences in Fgf-regulated *Mesp2* expression in the presomites between the littermate control and mutant embryos were observed.

We next examined whether conditional ablation of *Lrp6* in the dorsal neural folds affects additional critical genes, such as T-box, Fgf and Wnt genes in the tailbud signaling center, which are required for caudal body axis formation (Gofflot et al., 1997) and may also play a role in caudal neural tube closure. Wholemount *in situ* hybridization demonstrated that the expression patterns of brachyury (*T*) (Wilkinson et al., 1990), *Tbx6* (Chapman and Papaioannou, 1998), *Wnt1* (Parr et al., 1993) and *Wnt5a* (Yamaguchi et al., 1999) were not apparently altered in *Lrp6*-cKO tailbuds, dorsal neural folds or related caudal tissues at E9.5 (Fig. 3C-J). The expression patterns of *Fgf8*, *Fgf17* and *Fgf18* (Maruoka et al., 1998) were also not altered in the mutant tailbuds or related tissues (Fig. 4A-F). *Mesp2*, modulated by Notch and Fgf signaling (Niwa et al., 2011), is restrictively expressed in the presomites (Takahashi et al., 2000) and remained unaffected in E9.5 *Lrp6*-cKOs (Fig. 4G,H). These results suggest that the tailbud signaling center and new somite formation are not altered in the *Lrp6*-cKOs and might not contribute to PNP closure.

### Unchanged PCP signaling, proliferation and apoptosis in the dorsal PNPs of *Pax3Cre;Lrp6*-cKO mutants

**Fig. 5.**
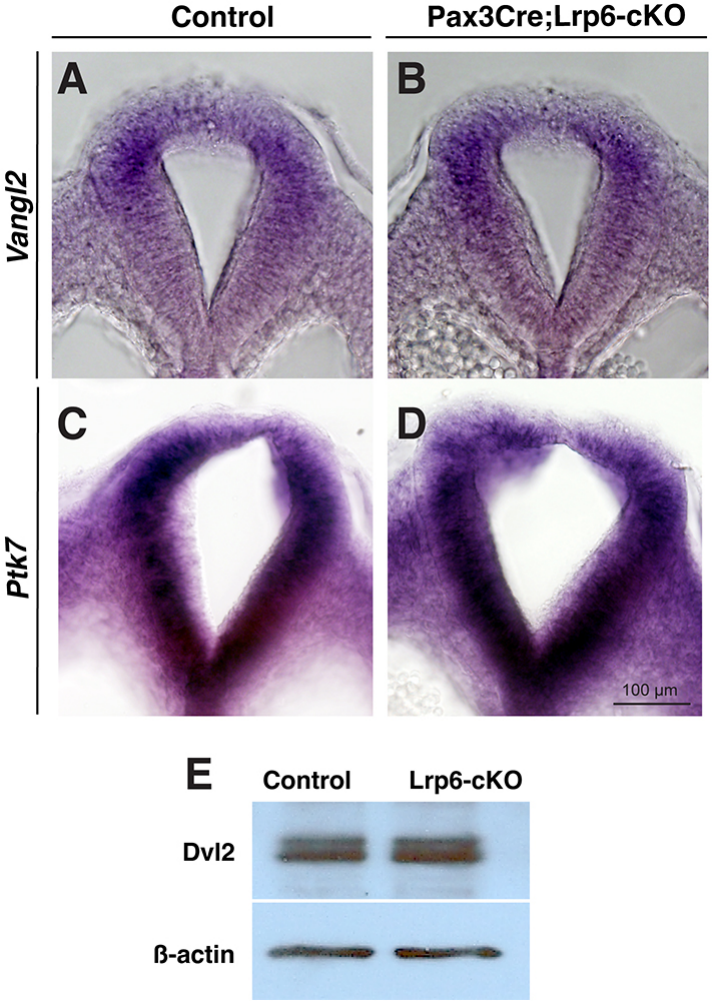
**Noncanonical Wnt/PCP signaling activities in littermate controls and *Pax3-Cre;Lrp6*-cKOs at E9.5.** (A-D) Transverse sections after wholemount *in situ* hybridization show no obvious changes in *Vangl2* (A,B) and *Ptk7* (C,D) expression patterns at the PNP closure sites in normal control and mutant embryos. (E) Immunoblots show no differences in phosphorylated (higher band that is linked with PCP signaling) and nonphosphorylated (lower band) Dvl2 proteins between the control and mutant PNP samples.

**Fig. 6.**
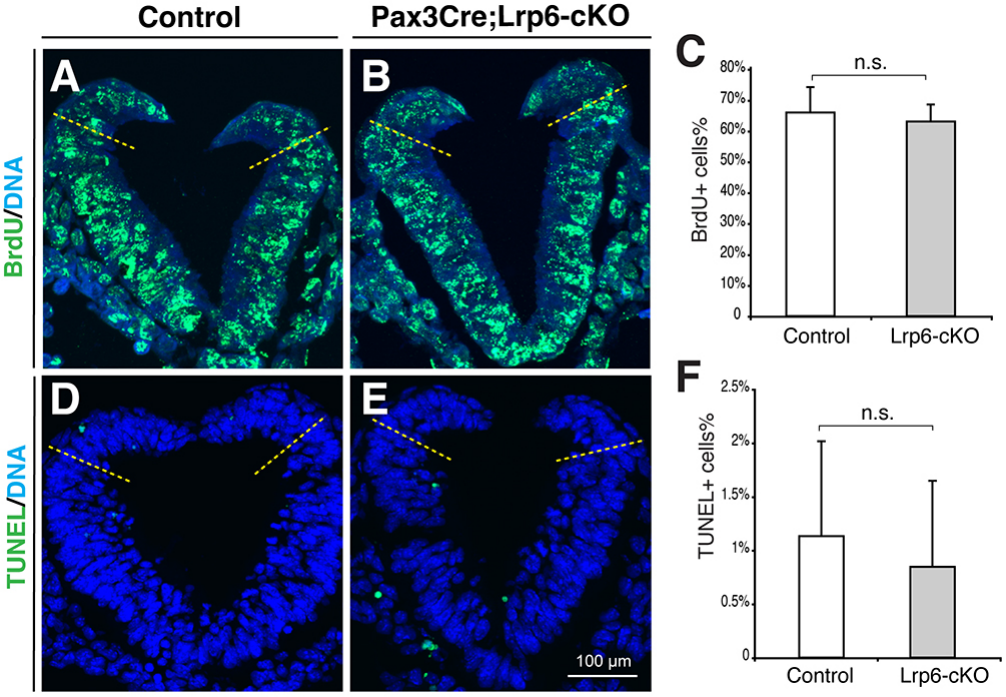
**Proliferation and apoptosis at the PNP closure sites of the littermate controls and *Pax3-Cre;Lrp6*-cKOs at E9.5.** (A-C) BrdU incorporation and detection experiments show no significant differences in proliferating cells in the dorsal PNPs above the dorsolateral hinge points (dashed lines in A,B, transverse PNP sections) between the control and mutant embryos. (D-F) TUNEL assays demonstrate no significant differences in apoptotic cells (green in D,E) in the dorsal PNPs between the control and mutant embryos. n.s., no statistical significance (*P*>0.05; unpaired, two-tailed Student's *t*-test).

To examine whether *Lrp6* deficiency in the dorsal neural folds affects noncanonical Wnt/PCP signaling, we first examined two representative genes, *Vangl2* (Kibar et al., 2001) and *Ptk7* (Lu et al., 2004), which are required for PCP signaling and neural tube closure. The expression of these genes was unaltered in the dorsal neural folds of *Lrp6*-cKO mutants at E9.5 (Fig. 5A-D). Dvl2 is a mediator of canonical and noncanonical Wnt signaling pathways and is required for neural tube closure (Hamblet et al., 2002). Dvl2 phosphorylation does not trigger β-catenin signaling and can be detected by band shifts of immunoblots (Gonzalez-Sancho et al., 2004). We detected no changes in Dvl2 phosphorylation in PNP samples of *Lrp6*-cKO mutants (Fig. 5E). In addition, proliferation, as shown by bromodeoxyuridine (BrdU) incorporation assays, and apoptosis, as shown by terminal deoxynucleotidyl transferase dUTP nick-end labeling (TUNEL) assays, were unaffected in the dorsal neural folds of *Lrp6*-cKO mutants (Fig. 6). These results suggest that conditional inactivation of *Lrp6* in dorsal neural folds may not modify PCP signaling, proliferation or apoptosis during caudal neural tube closure.

### Genetic activation of β-catenin can rescue closure defects in *Pax3Cre;Lrp6*-cKO mutants

**Fig. 7.**
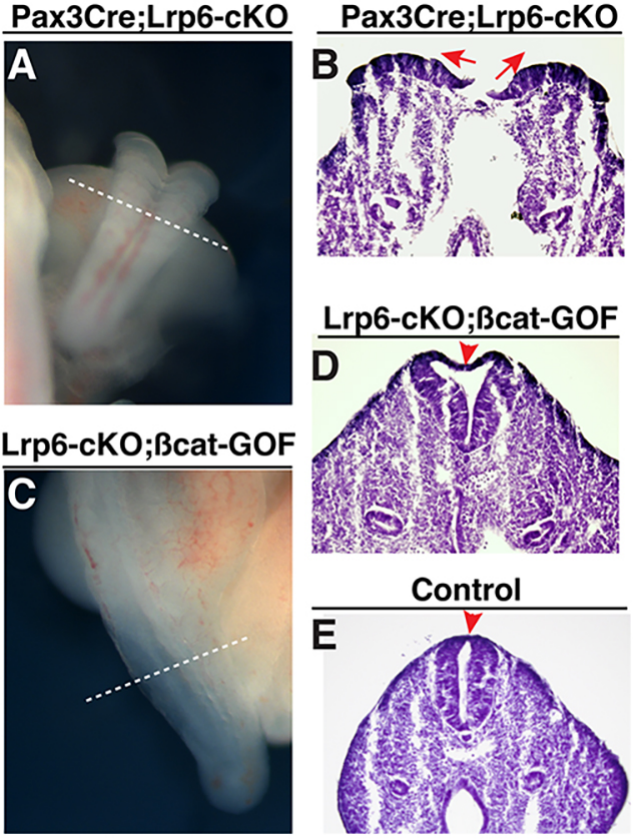
**Genetic rescue of PNP closure defects in the *Pax3-Cre;Lrp6*-cKOs by β-catenin gain-of-function (GOF).** (A,B) Failed PNP closure as shown in the dorsal–posterior view of an *Lrp6*-cKO embryo (A) and in a transverse PNP section (arrows in B, cut through the dashed line in A) at E10.5. (C,D) Rescued PNP closure as shown in the dorsal–posterior view of an *Lrp6*-cKO*;*β-catenin-GOF embryo (A) and in a transverse PNP section that shows abnormally widened but closed dorsal PNP (arrowhead in D, cut through the dashed line in C) at E10.5. (E) A transverse PNP section of a littermate control embryo shows normally closed PNP at the dorsal midline (arrowhead in E) at E10.5.

To verify that Lrp6 acts through β-catenin to regulate PNP closure, we tested genetic rescue experiments by gain-of-function (GOF) of β-catenin in *Lrp6*-cKO mutants. *Ctnnb1^flox(ex3)^* mice were used, in which the conditional removal of the floxed exon 3 (that encodes a phosphorylation site for β-catenin protein degradation) will stabilize β-catenin, leading to constitutive activation of canonical Wnt signaling (Harada et al., 1999). The results indicated rescued PNP closure in the compound mutants of *Pax3Cre;Lrp6*-cKO*;*β-catenin-GOF mutants (five out five embryos) at E10.5 (Fig. 7). However, the dorsal midline region and roof plate were markedly widened in the mutants, suggesting a side effect of constitutive activation of β-catenin signaling in the dorsal neural folds.

### Maternal supplementation of Wnt/β-catenin signaling agonists reduces the frequency and severity of spinal NTDs in *Pax3Cre;Lrp6*-cKO mutants

**Fig. 8.**
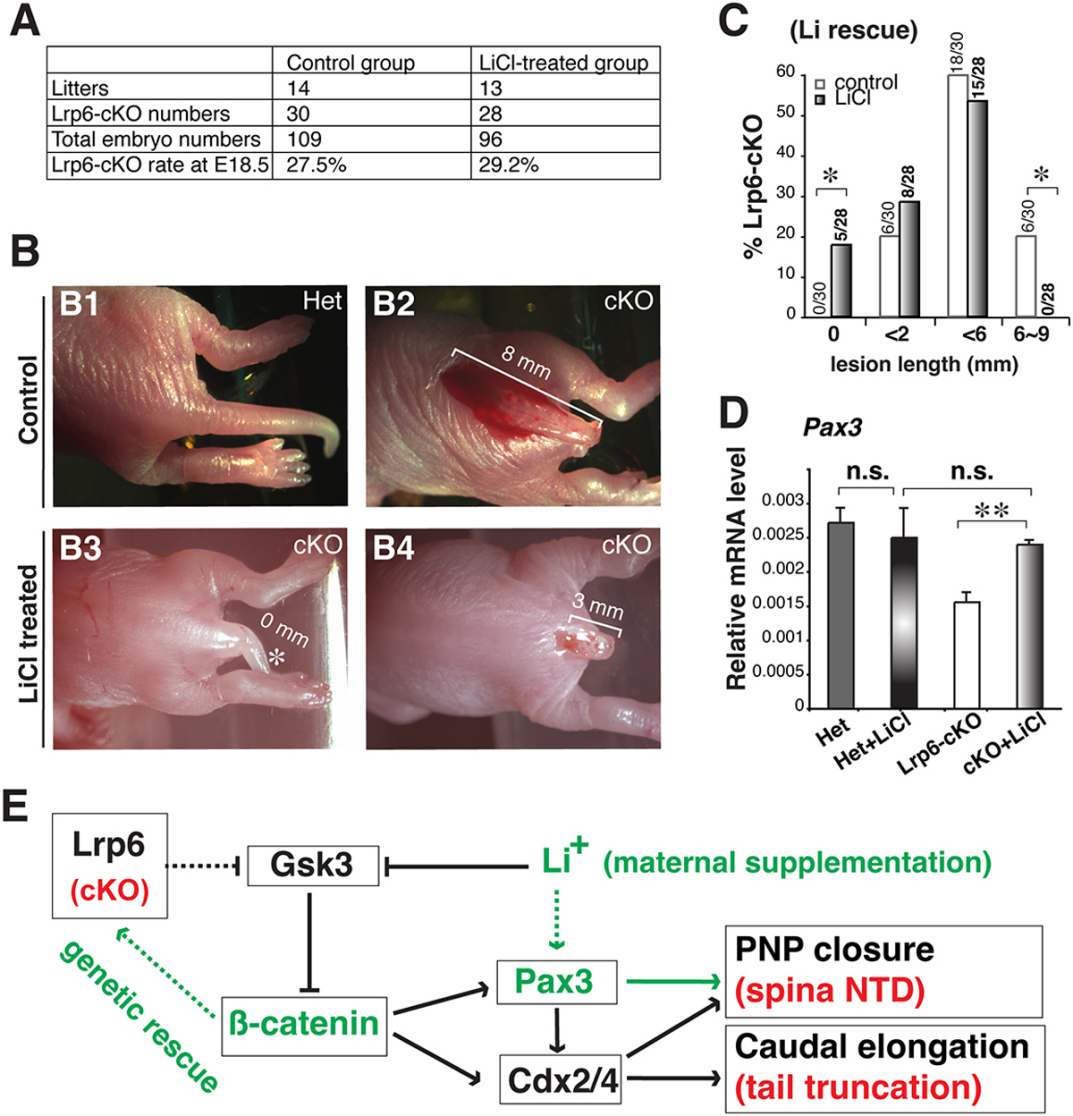
**Pharmaceutical intervention of spinal NTDs in *Pax3-Cre;Lrp6*-cKOs by maternal supplementation of a Wnt/β-catenin signaling agonist.** (A) The embryo numbers and *Lrp6*-cKO ratios are not significantly different between the control and lithium-treated groups at E18.5 as compared with the expected Mendelian ratio (25% cKOs) (*P*>0.05, chi-square test). (B) Dorsal–caudal body views of a double heterozygous (Het) *Pax3^Cre^;Lrp6^flox/+^* embryo that shows no NTD and with normal tail (B1), an *Lrp6*-cKO embryo in the control group that shows the severest lumbosacral NTD (with ∼8 mm lesion length, bracket in B2), an *Lrp6*-cKO embryo treated with lithium that shows a fully closed or rescued spinal cord (0 mm lesion length in B3, asterisk shows partially rescued tail growth), and a mutant embryo treated with lithium that shows milder NTD (with 3 mm lesion length in B4) at E18.5. (C) Rescue effects of spinal NTDs in Lrp6-cKOs examined at E18.5 after maternal supplementation of lithium chloride (LiCl) from E7.5 to E9.5. The lesion lengths (mm) were measured under a microscope. *P=0.02 (Fisher exact test); after all samples combined and averaged in each group, *P*=0.01 (unpaired, two-tailed Student's *t*-test). (D) RT-qPCR results demonstrate significant restoration of *Pax3* mRNA in the lithium-treated *Lrp6*-cKO PNPs at E9.5. n.s., no statistical significance (*P*>0.05); **P<0.01 (unpaired, two-tailed Student's *t*-test). (E) Illustrative summary of Lrp6-mediated β-catenin–Pax3/Cdx2/Cdx4 signaling underlying PNP closure/elongation and spinal NTD; the latter can be rescued by either genetic activation of β-catenin or maternal supplementation of lithium ion, which stabilizes intracellular β-catenin by inhibiting Gsk3 in the canonical Wnt signaling pathway, thus restoring a key downstream transcription factor *Pax3* in Lrp6-deficient PNPs. Red font, mutants and phenotypes; green font and arrows, genetic or pharmacological rescues demonstrated in the current study.

The finding that defective Wnt/β-catenin signaling pathway is essentially related to spinal NTDs provides a significant translational implication for NTD intervention. We therefore attempted to treat spinal NTDs in *Lrp6*-cKOs through maternal supplementation of lithium ion, a commonly used psychotherapeutic medication and well-known Wnt signaling agonist acting through inhibition of intracellular β-catenin degradation (Cohen and Goedert, 2004; Meijer et al., 2004). After intraperitoneal injections of lithium chloride (LiCl) solution to the pregnant females at E7.5-E9.5, ∼18% (5/28) of the *Lrp6*-cKO mice had completely closed neural tubes and elongated tails, as determined at E18.5, which were not observed in the control group of *Lrp6*-cKOs treated with the NaCl placebo (0/30 *Lrp6*-cKOs) (Fig. 8A-C). Conversely, 20% (6/30) of the *Lrp6*-cKOs in the control group exhibited severe closure defects up to the lumbar level, which were not observed in the LiCl-treated *Lrp6*-cKOs (0/28). These results demonstrate effective intervention of spinal NTDs in *Lrp6*-cKOs by lithium treatment. Lesions of variable lengths at the sacrococcygeal levels were also observed in both the LiCl-treated and placebo groups of *Lrp6*-cKOs, suggesting that environmental or other factors may affect canonical Wnt signaling activity and NTD severity. After lithium treatment, *Pax3* expression in the neural folds of *Lrp6*-cKOs was restored (Fig. 8D).

Together, these results elucidate the essential roles of the Lrp6-mediated canonical Wnt/β-catenin signaling pathway in murine caudal neural tube closure, which may provide significant clues to both the etiology and prevention of human NTDs.

## DISCUSSION

### Lrp6-mediated Wnt/β-catenin signaling during neural tube closure

This study reveals the essential role of Lrp6-mediated Wnt/β-catenin signaling in caudal neural tube closure. Lrp6 is a Wnt co-receptor acting upstream of β-catenin in the canonical pathway. We found fully penetrant spinal NTDs in *Pax3Cre;Lrp6*-cKO mutants, which is phenotypically consistent with *Pax3Cre;*β-catenin-cKO mutants, as demonstrated in our previous study (Zhao et al., 2014), suggesting a consistent role of Lrp6 and β-catenin in caudal neural tube closure. At the molecular signaling level, we observed diminished expression of several genes encoding transcription factors, including *Pax3*, *Cdx2*, *Cdx4* and *Msx1*, in the dorsal neural folds of *Lrp6*-cKO mutants, which were identically downregulated in the β-catenin-cKO neural folds, further suggesting that Lrp6 and β-catenin act in the same signaling cascade to regulate critical downstream target genes during caudal neural tube closure. Our genetic rescue experiments revealed that constitutively active β-catenin in the dorsal neural folds can rescue closure defects in *Lrp6*-cKO embryos, which firmly validates that Lrp6 acts through β-catenin to promote caudal neural tube closure. Moreover, maternal supplementation of an agonist of the Wnt/β-catenin signaling could reduce the frequency and severity of NTDs in *Lrp6*-cKO mutants, further substantiating the essential roles of Lrp6-mediated Wnt/β-catenin signaling in caudal neural tube closure.

We employed *Pax3^Cre^* knock-in mice for conditional gene-targeting analyses of both *Lrp6* and β-catenin, which generated consistent phenotypic and mechanistic results. Our genetic fate-mapping experiments demonstrated that *Pax3^Cre^* became activated in the border region between the non-neural surface ectoderm and neural plate in the pending closure sites of dorsal neural folds at E8.5. It has been demonstrated that conditional removal of β-catenin using *Grhl3^Cre^* in these border cells also causes spinal NTDs (Kimura-Yoshida et al., 2015). However, it remains unclear whether *Pax3^Cre^* and *Grhl3^Cre^* activities overlap in the same border cells during primary neurulation. We recently demonstrated that Grhl3 regulates dynamic movements of the non-neural surface ectodermal border cells for multicellular rosette formation and convergent cellular protrusions to promote caudal neural tube closure (Zhou et al., 2020). Conditional gene-targeting analyses of *Lrp6* using *Grhl3^Cre^* may clarify whether Lrp6-mediated Wnt/β-catenin signaling is also activated in these border cells as a modulator of their molecular properties and cellular dynamics for neural tube closure.

### Lrp6 and noncanonical Wnt signaling during neurulation

Lrp6 may exert a key role in the signaling interactions between canonical and noncanonical Wnt pathways. Loss-of-function (LOF) of Lrp6-mediated Wnt/β-catenin signaling may affect noncanonical Wnt/PCP signaling directly or indirectly. Our results showed that *Lrp6* deficiency did not change the expression patterns of two representative NTD-associated PCP signaling genes, *Vangl2* and *Ptk7*, in the mutant PNP, suggesting that Lrp6-mediated Wnt/β-catenin signaling does not directly regulate transcriptional activation of these PCP signaling genes. We also showed that *Lrp6* deficiency did not change the Dvl2 phosphorylation level. Because Dvl2 phosphorylation is independent of Lrp5/Lrp6 co-receptors and does not stabilize β-catenin (Gonzalez-Sancho et al., 2004), it is an excellent indicator of altered noncanonical Wnt signaling. It has been reported that different Wnts may signal through different co-receptors, Lrp6 for canonical signaling and Ror1/Ror2 for noncanonical signaling, and that canonical and noncanonical Wnts may exert reciprocal inhibition by competition for Fzd binding (Grumolato et al., 2010). Interestingly, an early study demonstrated that either LOF or GOF of *Lrp6* disrupted the convergent extension that is a hallmark of PCP signaling during *Xenopus* gastrulation (Tahinci et al., 2007). The authors showed that an intracellular domain of Lrp6 can inhibit Wnt/PCP signaling by unknown mechanisms, while potentiating Wnt/β-catenin signaling. Thus, Lrp6 may serve as a molecular switch from noncanonical to canonical Wnt signaling (Tahinci et al., 2007). Nevertheless, it remains unknown whether or how the noncanonical inhibitory function of this intracellular domain of Lrp6 contributes to neural tube closure. Another study reported that the extracellular domains of Lrp6 (or its homolog Lrp5) can also inhibit noncanonical Wnt signaling *in vitro* (Bryja et al., 2009). These authors further showed that *Wnt5a* deficiencies partially rescued heart defects and fully rescued the partially penetrant cranial NTD exencephaly in *Lrp6-*null mutants, suggesting that GOF of noncanonical Wnt signaling is the cause of these defects in *Lrp6*-null mutants (Bryja et al., 2009). However, *Wnt5a* deficiencies had no rescue effects on the fully penetrant spinal NTDs of *Lrp6*-null mutants, suggesting distinctly different mechanisms underlying cranial and spinal NTDs in *Lrp6*-deficient mutants. Above all, it will be important to determine whether upregulation of the Wnt/PCP signaling activities can indeed cause NTDs.

In contrast, the hypermorphic *Lrp6^crooked tail^* homozygous embryos exhibit cranial NTDs with defective RhoA signaling and apical–basal cell polarity, suggesting a defective noncanonical Wnt signaling mechanism (Carter et al., 2005). The compound mutants of another hypermorphic *Lrp6^Skax2^* homozygous combined with heterozygous *Vangl2^Lp^* exhibit spinal NTDs (Allache et al., 2014), reiterating a defective noncanonical Wnt signaling mechanism in these hypermorphic Lrp6 mutants. Nevertheless, it remains unknown whether or how noncanonical Wnt signaling is altered and may contribute to NTDs in *Lrp6*-deficient mutants and its contribution to NTD incidence. Mutants deficient in the PCP signaling gene *Ptk7* exhibit the severest NTD, craniorachischisis (Lu et al., 2004). Unexpectedly, a later study demonstrated that Ptk7 modulates both canonical and noncanonical Wnt signaling through physical interaction and stabilization of Lrp6 proteins (Bin-Nun et al., 2014). Therefore, Ptk7 or related PCP signaling may actually act upstream of Lrp6-mediated Wnt/β-catenin signaling, suggesting complex signaling crosstalk between the canonical and noncanonical Wnt pathways during neural tube closure.

### NTD intervention by targeting Lrp6-mediated Wnt/β-catenin signaling

The current study showed that maternal supplementation of LiCl during a relatively short period (from E7.5 to E9.5) can reduce the frequency and severity of spinal NTDs, with ∼18% of *Lrp6*-cKO mutants exhibiting completely closed neural tubes and an additional 20% with reduced NTD lesion sizes in the rescue group. Lithium inhibits Gsk3 enzymic activities (Klein and Melton, 1996) to stabilize β-catenin in the canonical Wnt signaling pathway (Liu et al., 2002). Thus, the results of our NTD rescue experiments by lithium support the role of Lrp6-mediated Wnt/β-catenin signaling in PNP closure and may also provide a translational implication for NTD intervention using small-molecule agonists of Wnt/β-catenin signaling (Fig. 8E). There are several other Gsk3 inhibitors that can be used for therapeutic treatments (Cohen and Goedert, 2004; Meijer et al., 2004), including the small-molecule CHIR99021 and 6-bromoindirubin-3′-oxime (BIO), which have been used widely as potent Wnt agonists (Sato et al., 2004; Silva et al., 2008). It is important to note that Gsk3 inhibitors are not specific to Wnt signaling and have broad functions, such as in insulin signaling, NFAT signaling and Hedgehog signaling (Cline et al., 2002; Crabtree and Olson, 2002; Price and Kalderon, 2002; Ring et al., 2003). Regardless of these broad effects, our results showed that lithium supplementation reduced the frequency and severity of NTDs in *Lrp6*-deficient mutants by restoring the expression of *Pax3*, which is a key downstream target gene of Wnt/β-catenin signaling. As demonstrated in our previous work, genetic activation of *Pax3* can rescue spinal NTDs in the conditional β-catenin mutants (Zhao et al., 2014). Intriguingly, *p53* (also known as *Trp53*) LOF by genetic or pharmacological approaches can rescue NTDs in *Pax3* mutants, suggesting a role of apoptosis in the development of NTDs (Pani et al., 2002). We did not detect significantly altered apoptosis in *Lrp6*-deficient mutants, but it would be important to test whether *p53* LOF can rescue NTDs in *Lrp6*-deficient mutants as well. Conversely, severe midline defects, including cranial and spinal NTDs and midline facial clefts, occurred in mice with triple knockout of the intrinsic apoptotic genes *Bax*, *Bak* (also known as *Bak1*) and *Bok* (Ke et al., 2018), demonstrating that developmental apoptosis is required for normal neural tube closure and related midline fusion processes. Thus, completely inhibiting apoptosis seems inappropriate for preventing NTDs.

In summary, this study demonstrated that conditional ablation of *Lrp6* in dorsal neural folds resulted in spinal NTDs due to diminished canonical Wnt/β-catenin signaling and downstream target genes. This could be rescued either by genetic activation or pharmacological stabilization of β-catenin *in vivo* (Fig. 8E), indicating LRP6-mediated Wnt/β-catenin signaling as a novel target for intervention for NTDs in humans.

## MATERIALS AND METHODS

### Animals

The *Lrp6^flox^* mice for conditional gene-targeting analyses have previously been described (Zhou et al., 2010). The *Pax3^Cre^* knock-in mice (Engleka et al., 2005), *Rosa26-lacZ* mice (Soriano, 1999), and canonical Wnt/β-catenin signaling reporter lines of *BATgal* (Maretto et al., 2003) and *TOPgal* (DasGupta and Fuchs, 1999) mice donated by different investigators were obtained through The Jackson Laboratory. *Ctnnb1^flox(ex3)^* mice (MGI:1858008, gift from M. Taketo, Kyoto University, Kyoto, Japan) (Harada et al., 1999) were used for genetic rescue by conditional activation of β-catenin. These mouse strains were maintained on a C57BL/6J or a mixed B6;129 background. All mice were housed in the vivarium at the University of California, Davis (Davis, CA, USA). Pregnant, timed-mated mice were euthanized prior to cesarean section. Noon of the conception day was designated as E0.5. All research procedures using mice were approved by the University of California, Davis Animal Care and Use Committee and conformed to National Institutes of Health guidelines.

### X-gal staining and wholemount *in situ* hybridization

Mouse embryos were fixed in 1% paraformaldehyde (PFA) for ∼30 min on ice and processed for X-gal staining as previously described (Song et al., 2009; Wang et al., 2011; Zhao et al., 2014). Embryos fixed in 4% PFA overnight at 4°C were processed for wholemount *in situ* hybridization using digoxigenin-labeled antisense RNA probes as previously described (Zhao et al., 2014). Antisense RNA probes were synthesized based on sequence information provided by the Allen Brain Atlas (https://portal.brain-map.org/). At least three mutants and three littermate control (Cre-lacking or double heterozygous) embryos were used for each *in situ* experiment, which showed consistent results.

### Maternal administration of LiCl

*In vivo* stimulation of the Wnt/β-catenin signaling pathway by LiCl was carried out as previously described with minor modifications (Song et al., 2009; Tian et al., 2010). Pregnant females were injected intraperitoneally with 200 mg/kg LiCl or an equivalent dose of a NaCl control solution on E7.5, E8.5 and E9.5. Embryos were collected at E18.5 for phenotype analysis or at E9.5 (1 h after the third-day injection) for real-time reverse transcription quantitative PCR (RT-qPCR) analyses.

### RNA isolation and real-time RT-qPCR

Total RNAs were isolated from the caudal neural folds and pooled from five E9.5 embryos of the mutant or control groups. Heterozygous *Pax3^Cre/+^* embryos were used as the control. After reverse-transcription, real-time PCR was carried as described in our previous publications (Song et al., 2009; Zhao et al., 2014). The mRNA level of *Pax3* was normalized to the mRNA level of glyceraldehyde-3-phosphate dehydrogenase (*Gapdh*) to allow for relative comparisons among different experimental groups using the ΔΔC_t_ method.

### BrdU labeling, immunohistochemistry and TUNEL assays

Acute BrdU labeling was performed by intraperitoneal injection of BrdU at 100 mg/kg body weight to the pregnant mice 1 h prior to sampling. Immunohistochemistry was carried out on paraffin or frozen sections using primary antibodies against BrdU (1:100; M0744, Dako) and Alexa Fluor-conjugated secondary antibodies (1:400; Molecular Probes). TUNEL assays were performed using the Dead End Fluorometric TUNEL System (Promega) as detailed by the manufacturer. The total numbers of BrdU- or TUNEL-positive cells were counted in the dorsal PNP above the dorsolateral hinge points on each confocal micrograph (Fig. 6) then divided by the total cell numbers on the same areas to obtain the percentages of the positive cells. In each experiment, at least three sections from three mutants or three littermate controls at the same age were counted and averaged for statistical analyses.

### Immunoblotting

Immunoblotting was carried out according to the standard protocol (Wang et al., 2016). Protein samples were pooled from the caudal part of five embryos of E9.5 control or *Pax3Cre;Lrp6*-cKO embryos. Primary antibodies against Dvl2 (1:200; sc-13974, Santa Cruz Biotechnology) and β-actin (1:5000; sc-1616, Santa Cruz Biotechnology) were used.

### Statistical analyses

At least three littermate controls and three mutant embryos were used for each statistical evaluation. Significances were assessed by unpaired, two-tailed Student's *t*-test or pairwise comparison (one-way ANOVA) when appropriate. In all cases, *P*≤0.05 was considered statistically significant.

## References

[DMM049517C1] Allache, R., Lachance, S., Guyot, M. C., De Marco, P., Merello, E., Justice, M. J., Capra, V. and Kibar, Z. (2014). Novel mutations in Lrp6 orthologs in mouse and human neural tube defects affect a highly dosage-sensitive Wnt non-canonical planar cell polarity pathway. *Hum. Mol. Genet.* 23, 1687-1699. 10.1093/hmg/ddt558 24203697 PMC3943515

[DMM049517C2] Alrefaei, A. F. and Abu-Elmagd, M. (2022). LRP6 receptor plays essential functions in development and human diseases. *Genes (Basel)* 13, 120. 10.3390/genes13010120 35052459 PMC8775365

[DMM049517C3] Au, K. S., Ashley-Koch, A. and Northrup, H. (2010). Epidemiologic and genetic aspects of spina bifida and other neural tube defects. *Dev. Disabil. Res. Rev.* 16, 6-15. 10.1002/ddrr.93 20419766 PMC3053142

[DMM049517C4] Bin-Nun, N., Lichtig, H., Malyarova, A., Levy, M., Elias, S. and Frank, D. (2014). PTK7 modulates Wnt signaling activity via LRP6. *Development* 141, 410-421. 10.1242/dev.095984 24353057

[DMM049517C5] Bryja, V., Andersson, E. R., Schambony, A., Esner, M., Bryjova, L., Biris, K. K., Hall, A. C., Kraft, B., Cajanek, L., Yamaguchi, T. P. et al. (2009). The extracellular domain of Lrp5/6 inhibits noncanonical Wnt signaling in vivo. *Mol. Biol. Cell* 20, 924-936. 10.1091/mbc.e08-07-0711 19056682 PMC2633404

[DMM049517C6] Carter, M., Chen, X., Slowinska, B., Minnerath, S., Glickstein, S., Shi, L., Campagne, F., Weinstein, H. and Ross, M. E. (2005). Crooked tail (Cd) model of human folate-responsive neural tube defects is mutated in Wnt coreceptor lipoprotein receptor-related protein 6. *Proc. Natl. Acad. Sci. USA* 102, 12843-12848. 10.1073/pnas.0501963102 16126904 PMC1200260

[DMM049517C7] Centers for Disease Control and Prevention (CDC) (2004). Spina bifida and anencephaly before and after folic acid mandate--United States, 1995-1996 and 1999-2000. *MMWR Morb. Mortal. Wkly. Rep.* 53, 362-365. 15129193

[DMM049517C8] Chapman, D. L. and Papaioannou, V. E. (1998). Three neural tubes in mouse embryos with mutations in the T-box gene Tbx6. *Nature* 391, 695-697. 10.1038/35624 9490412

[DMM049517C9] Cline, G. W., Johnson, K., Regittnig, W., Perret, P., Tozzo, E., Xiao, L., Damico, C. and Shulman, G. I. (2002). Effects of a novel glycogen synthase kinase-3 inhibitor on insulin-stimulated glucose metabolism in Zucker diabetic fatty (fa/fa) rats. *Diabetes* 51, 2903-2910. 10.2337/diabetes.51.10.2903 12351425

[DMM049517C10] Cohen, P. and Goedert, M. (2004). GSK3 inhibitors: development and therapeutic potential. *Nat. Rev. Drug Discov.* 3, 479-487. 10.1038/nrd1415 15173837

[DMM049517C11] Copp, A. J., Greene, N. D. and Murdoch, J. N. (2003). The genetic basis of mammalian neurulation. *Nat. Rev. Genet.* 4, 784-793. 10.1038/nrg1181 13679871

[DMM049517C12] Copp, A. J., Stanier, P. and Greene, N. D. (2013). Neural tube defects: recent advances, unsolved questions, and controversies. *Lancet Neurol.* 12, 799-810. 10.1016/S1474-4422(13)70110-8 23790957 PMC4023229

[DMM049517C13] Crabtree, G. R. and Olson, E. N. (2002). NFAT signaling: choreographing the social lives of cells. *Cell* 109 Suppl, S67-S79. 10.1016/S0092-8674(02)00699-2 11983154

[DMM049517C14] DasGupta, R. and Fuchs, E. (1999). Multiple roles for activated LEF/TCF transcription complexes during hair follicle development and differentiation. *Development* 126, 4557-4568. 10.1242/dev.126.20.4557 10498690

[DMM049517C15] De Marco, P., Merello, E., Cama, A., Kibar, Z. and Capra, V. (2011). Human neural tube defects: genetic causes and prevention. *Biofactors* 37, 261-268. 10.1002/biof.170 21674647

[DMM049517C16] De Marco, P., Merello, E., Piatelli, G., Cama, A., Kibar, Z. and Capra, V. (2014). Planar cell polarity gene mutations contribute to the etiology of human neural tube defects in our population. *Birth Defects Res. A Clin. Mol. Teratol* 100, 633-641. 10.1002/bdra.23255 24838524

[DMM049517C17] Engleka, K. A., Gitler, A. D., Zhang, M., Zhou, D. D., High, F. A. and Epstein, J. A. (2005). Insertion of Cre into the Pax3 locus creates a new allele of Splotch and identifies unexpected Pax3 derivatives. *Dev. Biol.* 280, 396-406. 10.1016/j.ydbio.2005.02.002 15882581

[DMM049517C18] Epstein, D. J., Vekemans, M. and Gros, P. (1991). Splotch (Sp2H), a mutation affecting development of the mouse neural tube, shows a deletion within the paired homeodomain of Pax-3. *Cell* 67, 767-774. 10.1016/0092-8674(91)90071-6 1682057

[DMM049517C19] Foerst-Potts, L. and Sadler, T. W. (1997). Disruption of Msx-1 and Msx-2 reveals roles for these genes in craniofacial, eye, and axial development. *Dev. Dyn.* 209, 70-84. 10.1002/(SICI)1097-0177(199705)209:1<70::AID-AJA7>3.0.CO;2-U 9142497

[DMM049517C20] Gofflot, F., Hall, M. and Morriss-Kay, G. M. (1997). Genetic patterning of the developing mouse tail at the time of posterior neuropore closure. *Dev. Dyn.* 210, 431-445. 10.1002/(SICI)1097-0177(199712)210:4<431::AID-AJA7>3.0.CO;2-H 9415428

[DMM049517C21] Gonzalez-Sancho, J. M., Brennan, K. R., Castelo-Soccio, L. A. and Brown, A. M. (2004). Wnt proteins induce dishevelled phosphorylation via an LRP5/6- independent mechanism, irrespective of their ability to stabilize beta-catenin. *Mol. Cell. Biol.* 24, 4757-4768. 10.1128/MCB.24.11.4757-4768.2004 15143170 PMC416421

[DMM049517C22] Goulding, M. D., Chalepakis, G., Deutsch, U., Erselius, J. R. and Gruss, P. (1991). Pax-3, a novel murine DNA binding protein expressed during early neurogenesis. *EMBO J.* 10, 1135-1147. 10.1002/j.1460-2075.1991.tb08054.x 2022185 PMC452767

[DMM049517C23] Gray, J. D., Kholmanskikh, S., Castaldo, B. S., Hansler, A., Chung, H., Klotz, B., Singh, S., Brown, A. M. and Ross, M. E. (2013). LRP6 exerts non-canonical effects on Wnt signaling during neural tube closure. *Hum. Mol. Genet.* 22, 4267-4281. 10.1093/hmg/ddt277 23773994 PMC3792688

[DMM049517C24] Greene, N. D. and Copp, A. J. (2005). Mouse models of neural tube defects: investigating preventive mechanisms. *Am. J. Med. Genet. C Semin. Med. Genet.* 135C, 31-41. 10.1002/ajmg.c.30051 15800852

[DMM049517C25] Greene, N. D. and Copp, A. J. (2014). Neural tube defects. *Annu. Rev. Neurosci.* 37, 221-242. 10.1146/annurev-neuro-062012-170354 25032496 PMC4486472

[DMM049517C26] Grumolato, L., Liu, G., Mong, P., Mudbhary, R., Biswas, R., Arroyave, R., Vijayakumar, S., Economides, A. N. and Aaronson, S. A. (2010). Canonical and noncanonical Wnts use a common mechanism to activate completely unrelated coreceptors. *Genes Dev.* 24, 2517-2530. 10.1101/gad.1957710 21078818 PMC2975928

[DMM049517C27] Hamblet, N. S., Lijam, N., Ruiz-Lozano, P., Wang, J., Yang, Y., Luo, Z., Mei, L., Chien, K. R., Sussman, D. J. and Wynshaw-Boris, A. (2002). Dishevelled 2 is essential for cardiac outflow tract development, somite segmentation and neural tube closure. *Development* 129, 5827-5838. 10.1242/dev.00164 12421720

[DMM049517C28] Harada, N., Tamai, Y., Ishikawa, T., Sauer, B., Takaku, K., Oshima, M. and Taketo, M. M. (1999). Intestinal polyposis in mice with a dominant stable mutation of the beta-catenin gene. *EMBO J.* 18, 5931-5942. 10.1093/emboj/18.21.5931 10545105 PMC1171659

[DMM049517C29] Harris, M. J. and Juriloff, D. M. (2007). Mouse mutants with neural tube closure defects and their role in understanding human neural tube defects. *Birth Defects Res. A Clin. Mol. Teratol* 79, 187-210. 10.1002/bdra.20333 17177317

[DMM049517C30] Harris, M. J. and Juriloff, D. M. (2010). An update to the list of mouse mutants with neural tube closure defects and advances toward a complete genetic perspective of neural tube closure. *Birth Defects Res. A Clin. Mol. Teratol* 88, 653-669. 10.1002/bdra.20676 20740593

[DMM049517C31] He, X., Semenov, M., Tamai, K. and Zeng, X. (2004). LDL receptor-related proteins 5 and 6 in Wnt/beta-catenin signaling: arrows point the way. *Development* 131, 1663-1677. 10.1242/dev.01117 15084453

[DMM049517C32] Jho, E. H., Zhang, T., Domon, C., Joo, C. K., Freund, J. N. and Costantini, F. (2002). Wnt/beta-catenin/Tcf signaling induces the transcription of Axin2, a negative regulator of the signaling pathway. *Mol. Cell. Biol.* 22, 1172-1183. 10.1128/MCB.22.4.1172-1183.2002 11809808 PMC134648

[DMM049517C33] Juriloff, D. M. and Harris, M. J. (2012). A consideration of the evidence that genetic defects in planar cell polarity contribute to the etiology of human neural tube defects. *Birth Defects Res. A Clin. Mol. Teratol* 94, 824-840. 10.1002/bdra.23079 23024041

[DMM049517C34] Ke, F. F. S., Vanyai, H. K., Cowan, A. D., Delbridge, A. R. D., Whitehead, L., Grabow, S., Czabotar, P. E., Voss, A. K. and Strasser, A. (2018). Embryogenesis and adult life in the absence of intrinsic apoptosis effectors BAX, BAK, and BOK. *Cell* 173, 1217-1230. 10.1016/j.cell.2018.04.036 29775594

[DMM049517C35] Kibar, Z., Vogan, K. J., Groulx, N., Justice, M. J., Underhill, D. A. and Gros, P. (2001). Ltap, a mammalian homolog of Drosophila Strabismus/Van Gogh, is altered in the mouse neural tube mutant Loop-tail. *Nat. Genet.* 28, 251-255. 10.1038/90081 11431695

[DMM049517C36] Kimura-Yoshida, C., Mochida, K., Ellwanger, K., Niehrs, C. and Matsuo, I. (2015). Fate specification of neural plate border by canonical Wnt Signaling and Grhl3 is crucial for neural tube closure. *EBioMedicine* 2, 513-527. 10.1016/j.ebiom.2015.04.012 26288816 PMC4535158

[DMM049517C37] Klein, P. S. and Melton, D. A. (1996). A molecular mechanism for the effect of lithium on development. *Proc. Natl. Acad. Sci. USA* 93, 8455-8459. 10.1073/pnas.93.16.8455 8710892 PMC38692

[DMM049517C38] Kokubu, C., Heinzmann, U., Kokubu, T., Sakai, N., Kubota, T., Kawai, M., Wahl, M. B., Galceran, J., Grosschedl, R., Ozono, K. et al. (2004). Skeletal defects in ringelschwanz mutant mice reveal that Lrp6 is required for proper somitogenesis and osteogenesis. *Development* 131, 5469-5480. 10.1242/dev.01405 15469977

[DMM049517C39] Lang, D., Lu, M. M., Huang, L., Engleka, K. A., Zhang, M., Chu, E. Y., Lipner, S., Skoultchi, A., Millar, S. E. and Epstein, J. A. (2005). Pax3 functions at a nodal point in melanocyte stem cell differentiation. *Nature* 433, 884-887. 10.1038/nature03292 15729346

[DMM049517C40] Lei, Y., Fathe, K., McCartney, D., Zhu, H., Yang, W., Ross, M. E., Shaw, G. M. and Finnell, R. H. (2015). Rare LRP6 variants identified in spina bifida patients. *Hum. Mutat.* 36, 342-349. 10.1002/humu.22750 25546815 PMC4361299

[DMM049517C41] Liu, C., Li, Y., Semenov, M., Han, C., Baeg, G. H., Tan, Y., Zhang, Z., Lin, X. and He, X. (2002). Control of beta-catenin phosphorylation/degradation by a dual-kinase mechanism. *Cell* 108, 837-847. 10.1016/S0092-8674(02)00685-2 11955436

[DMM049517C42] Lu, X., Borchers, A. G., Jolicoeur, C., Rayburn, H., Baker, J. C. and Tessier-Lavigne, M. (2004). PTK7/CCK-4 is a novel regulator of planar cell polarity in vertebrates. *Nature* 430, 93-98. 10.1038/nature02677 15229603

[DMM049517C43] MacDonald, B. T., Tamai, K. and He, X. (2009). Wnt/beta-catenin signaling: components, mechanisms, and diseases. *Dev. Cell* 17, 9-26. 10.1016/j.devcel.2009.06.016 19619488 PMC2861485

[DMM049517C44] Mao, B., Wu, W., Li, Y., Hoppe, D., Stannek, P., Glinka, A. and Niehrs, C. (2001). LDL-receptor-related protein 6 is a receptor for Dickkopf proteins. *Nature* 411, 321-325. 10.1038/35077108 11357136

[DMM049517C45] Maretto, S., Cordenonsi, M., Dupont, S., Braghetta, P., Broccoli, V., Hassan, A. B., Volpin, D., Bressan, G. M. and Piccolo, S. (2003). Mapping Wnt/beta-catenin signaling during mouse development and in colorectal tumors. *Proc. Natl. Acad. Sci. USA* 100, 3299-3304. 10.1073/pnas.0434590100 12626757 PMC152286

[DMM049517C46] Maruoka, Y., Ohbayashi, N., Hoshikawa, M., Itoh, N., Hogan, B. L. and Furuta, Y. (1998). Comparison of the expression of three highly related genes, Fgf8, Fgf17 and Fgf18, in the mouse embryo. *Mech. Dev.* 74, 175-177. 10.1016/S0925-4773(98)00061-6 9651520

[DMM049517C47] Meijer, L., Flajolet, M. and Greengard, P. (2004). Pharmacological inhibitors of glycogen synthase kinase 3. *Trends Pharmacol. Sci.* 25, 471-480. 10.1016/j.tips.2004.07.006 15559249

[DMM049517C48] Monsoro-Burq, A. H., Wang, E. and Harland, R. (2005). Msx1 and Pax3 cooperate to mediate FGF8 and WNT signals during Xenopus neural crest induction. *Dev. Cell* 8, 167-178. 10.1016/j.devcel.2004.12.017 15691759

[DMM049517C49] Niwa, Y., Shimojo, H., Isomura, A., Gonzalez, A., Miyachi, H. and Kageyama, R. (2011). Different types of oscillations in Notch and Fgf signaling regulate the spatiotemporal periodicity of somitogenesis. *Genes Dev.* 25, 1115-1120. 10.1101/gad.2035311 21632822 PMC3110950

[DMM049517C50] Pani, L., Horal, M. and Loeken, M. R. (2002). Rescue of neural tube defects in Pax-3-deficient embryos by p53 loss of function: implications for Pax-3- dependent development and tumorigenesis. *Genes Dev.* 16, 676-680. 10.1101/gad.969302 11914272 PMC155364

[DMM049517C51] Parr, B. A., Shea, M. J., Vassileva, G. and McMahon, A. P. (1993). Mouse Wnt genes exhibit discrete domains of expression in the early embryonic CNS and limb buds. *Development* 119, 247-261. 10.1242/dev.119.1.247 8275860

[DMM049517C52] Pinson, K. I., Brennan, J., Monkley, S., Avery, B. J. and Skarnes, W. C. (2000). An LDL-receptor-related protein mediates Wnt signalling in mice. *Nature* 407, 535-538. 10.1038/35035124 11029008

[DMM049517C53] Price, M. A. and Kalderon, D. (2002). Proteolysis of the Hedgehog signaling effector Cubitus interruptus requires phosphorylation by Glycogen Synthase Kinase 3 and Casein Kinase 1. *Cell* 108, 823-835. 10.1016/S0092-8674(02)00664-5 11955435

[DMM049517C54] Ring, D. B., Johnson, K. W., Henriksen, E. J., Nuss, J. M., Goff, D., Kinnick, T. R., Ma, S. T., Reeder, J. W., Samuels, I., Slabiak, T. et al. (2003). Selective glycogen synthase kinase 3 inhibitors potentiate insulin activation of glucose transport and utilization in vitro and in vivo. *Diabetes* 52, 588-595. 10.2337/diabetes.52.3.588 12606497

[DMM049517C55] Sato, N., Meijer, L., Skaltsounis, L., Greengard, P. and Brivanlou, A. H. (2004). Maintenance of pluripotency in human and mouse embryonic stem cells through activation of Wnt signaling by a pharmacological GSK-3-specific inhibitor. *Nat. Med.* 10, 55-63. 10.1038/nm979 14702635

[DMM049517C56] Shi, Z., Yang, X., Li, B. B., Chen, S., Yang, L., Cheng, L., Zhang, T., Wang, H. and Zheng, Y. (2018). Novel mutation of LRP6 identified in chinese han population links canonical WNT signaling to neural tube defects. *Birth Defects Res.* 110, 63-71. 10.1002/bdr2.1122 28960852

[DMM049517C57] Silva, J., Barrandon, O., Nichols, J., Kawaguchi, J., Theunissen, T. W. and Smith, A. (2008). Promotion of reprogramming to ground state pluripotency by signal inhibition. *PLoS Biol.* 6, e253. 10.1371/journal.pbio.0060253 18942890 PMC2570424

[DMM049517C58] Song, L., Li, Y., Wang, K., Wang, Y. Z., Molotkov, A., Gao, L., Zhao, T., Yamagami, T., Wang, Y., Gan, Q. et al. (2009). Lrp6-mediated canonical Wnt signaling is required for lip formation and fusion. *Development* 136, 3161-3371. 10.1242/dev.037440 19700620

[DMM049517C59] Song, L., Li, Y., Wang, K. and Zhou, C. J. (2010). Cardiac neural crest and outflow tract defects in Lrp6 mutant mice. *Dev. Dyn.* 239, 200-210. 10.1002/dvdy.22079 19705442

[DMM049517C60] Soriano, P. (1999). Generalized lacZ expression with the ROSA26 Cre reporter strain. *Nat. Genet.* 21, 70-71. 10.1038/5007 9916792

[DMM049517C61] Tahinci, E., Thorne, C. A., Franklin, J. L., Salic, A., Christian, K. M., Lee, L. A., Coffey, R. J. and Lee, E. (2007). Lrp6 is required for convergent extension during Xenopus gastrulation. *Development* 134, 4095-4106. 10.1242/dev.010272 17965054 PMC4428168

[DMM049517C62] Takahashi, Y., Koizumi, K., Takagi, A., Kitajima, S., Inoue, T., Koseki, H. and Saga, Y. (2000). Mesp2 initiates somite segmentation through the Notch signalling pathway. *Nat. Genet.* 25, 390-396. 10.1038/78062 10932180

[DMM049517C63] Tamai, K., Semenov, M., Kato, Y., Spokony, R., Liu, C., Katsuyama, Y., Hess, F., Saint-Jeannet, J. P. and He, X. (2000). LDL-receptor-related proteins in Wnt signal transduction. *Nature* 407, 530-535. 10.1038/35035117 11029007

[DMM049517C64] Tian, Y., Yuan, L., Goss, A. M., Wang, T., Yang, J., Lepore, J. J., Zhou, D., Schwartz, R. J., Patel, V., Cohen, E. D. et al. (2010). Characterization and in vivo pharmacological rescue of a Wnt2-Gata6 pathway required for cardiac inflow tract development. *Dev. Cell* 18, 275-287. 10.1016/j.devcel.2010.01.008 20159597 PMC2846539

[DMM049517C65] Tobin, M., Gunaji, R., Walsh, J. C. and Grice, G. P. (2019). A review of genetic factors underlying craniorachischisis and omphalocele: Inspired by a unique trisomy 18 case. *Am. J. Med. Genet. A* 179, 1642-1651. 10.1002/ajmg.a.61255 31184807

[DMM049517C66] Ukita, K., Hirahara, S., Oshima, N., Imuta, Y., Yoshimoto, A., Jang, C. W., Oginuma, M., Saga, Y., Behringer, R. R., Kondoh, H. et al. (2009). Wnt signaling maintains the notochord fate for progenitor cells and supports the posterior extension of the notochord. *Mech. Dev.* 126, 791-803. 10.1016/j.mod.2009.08.003 19720144 PMC2757446

[DMM049517C67] van Nes, J., de Graaff, W., Lebrin, F., Gerhard, M., Beck, F. and Deschamps, J. (2006). The Cdx4 mutation affects axial development and reveals an essential role of Cdx genes in the ontogenesis of the placental labyrinth in mice. *Development* 133, 419-428. 10.1242/dev.02216 16396910

[DMM049517C68] Wallingford, J. B. (2006). Planar cell polarity, ciliogenesis and neural tube defects. *Hum. Mol. Genet.* 15, R227-R234. 10.1093/hmg/ddl216 16987888

[DMM049517C69] Wallingford, J. B., Niswander, L. A., Shaw, G. M. and Finnell, R. H. (2013). The continuing challenge of understanding, preventing, and treating neural tube defects. *Science* 339, 1222002. 10.1126/science.1222002 23449594 PMC3677196

[DMM049517C70] Wang, Y., Song, L. and Zhou, C. J. (2011). The canonical Wnt/beta-catenin signaling pathway regulates Fgf signaling for early facial development. *Dev. Biol.* 349, 250-260. 10.1016/j.ydbio.2010.11.004 21070765

[DMM049517C71] Wang, Y., Stokes, A., Duan, Z., Hui, J., Xu, Y., Chen, Y., Chen, H. W., Lam, K. and Zhou, C. J. (2016). LDL receptor-related protein 6 modulates ret proto-oncogene signaling in renal development and cystic dysplasia. *J. Am. Soc. Nephrol.* 27, 417-427. 10.1681/ASN.2014100998 26047795 PMC4731110

[DMM049517C72] Wang, M., Marco, P., Capra, V. and Kibar, Z. (2019). Update on the role of the non-canonical Wnt/planar cell polarity pathway in neural tube defects. *Cells* 8, 1198. 10.3390/cells8101198 PMC6829399 31590237

[DMM049517C73] Wehrli, M., Dougan, S. T., Caldwell, K., O'Keefe, L., Schwartz, S., Vaizel-Ohayon, D., Schejter, E., Tomlinson, A. and DiNardo, S. (2000). arrow encodes an LDL-receptor-related protein essential for Wingless signalling. *Nature* 407, 527-530. 10.1038/35035110 11029006

[DMM049517C74] Wilkinson, D. G., Bhatt, S. and Herrmann, B. G. (1990). Expression pattern of the mouse T gene and its role in mesoderm formation. *Nature* 343, 657-659. 10.1038/343657a0 1689462

[DMM049517C75] Williams, J., Mai, C. T., Mulinare, J., Isenburg, J., Flood, T. J., Ethen, M., Frohnert, B. and Kirby, R. S., Centers for Disease Control and Prevention (CDC) (2015). Updated estimates of neural tube defects prevented by mandatory folic Acid fortification - United States, 1995-2011. *MMWR Morb. Mortal. Wkly. Rep.* 64, 1-5. 25590678 PMC4584791

[DMM049517C76] Yamaguchi, T. P., Bradley, A., McMahon, A. P. and Jones, S. (1999). A Wnt5a pathway underlies outgrowth of multiple structures in the vertebrate embryo. *Development* 126, 1211-1223. 10.1242/dev.126.6.1211 10021340

[DMM049517C77] Ybot-Gonzalez, P., Savery, D., Gerrelli, D., Signore, M., Mitchell, C. E., Faux, C. H., Greene, N. D. and Copp, A. J. (2007). Convergent extension, planar-cell-polarity signalling and initiation of mouse neural tube closure. *Development* 134, 789-799. 10.1242/dev.000380 17229766 PMC1839770

[DMM049517C78] Young, T., Rowland, J. E., van de Ven, C., Bialecka, M., Novoa, A., Carapuco, M., van Nes, J., de Graaff, W., Duluc, I., Freund, J. N. et al. (2009). Cdx and Hox genes differentially regulate posterior axial growth in mammalian embryos. *Dev. Cell* 17, 516-526. 10.1016/j.devcel.2009.08.010 19853565

[DMM049517C79] Zaganjor, I., Sekkarie, A., Tsang, B. L., Williams, J., Razzaghi, H., Mulinare, J., Sniezek, J. E., Cannon, M. J. and Rosenthal, J. (2016). Describing the prevalence of neural tube defects worldwide: a systematic literature review. *PLoS One* 11, e0151586. 10.1371/journal.pone.0151586 27064786 PMC4827875

[DMM049517C80] Zhao, T., Gan, Q., Stokes, A., Lassiter, R. N., Wang, Y., Chan, J., Han, J. X., Pleasure, D. E., Epstein, J. A. and Zhou, C. J. (2014). beta-catenin regulates Pax3 and Cdx2 for caudal neural tube closure and elongation. *Development* 141, 148-157. 10.1242/dev.101550 24284205 PMC3865756

[DMM049517C81] Zhou, C. J., Ji, Y., Reynolds, K., McMahon, M., Garland, M. A., Zhang, S., Sun, B., Gu, R., Islam, M., Liu, Y. et al. (2020). Non-neural surface ectodermal rosette formation and F-actin dynamics drive mammalian neural tube closure. *Biochem. Biophys. Res. Commun.* 526, 647-653. 10.1016/j.bbrc.2020.03.138 32248972 PMC7210071

[DMM049517C82] Zhou, C. J., Pinson, K. I. and Pleasure, S. J. (2004). Severe defects in dorsal thalamic development in low-density lipoprotein receptor-related protein-6 mutants. *J. Neurosci.* 24, 7632-7639. 10.1523/JNEUROSCI.2123-04.2004 15342729 PMC6729615

[DMM049517C83] Zhou, C. J., Molotkov, A., Song, L., Li, Y., Pleasure, D. E., Pleasure, S. J. and Wang, Y. Z. (2008). Ocular coloboma and dorsoventral neuroretinal patterning defects in Lrp6 mutant eyes. *Dev. Dyn.* 237, 3681-3689. 10.1002/dvdy.21770 18985738 PMC2727282

[DMM049517C84] Zhou, C. J., Wang, Y. Z., Yamagami, T., Zhao, T., Song, L. and Wang, K. (2010). Generation of Lrp6 conditional gene-targeting mouse line for modeling and dissecting multiple birth defects/congenital anomalies. *Dev. Dyn.* 239, 318-326. 10.1002/dvdy.22054 19653321

[DMM049517C85] Zohn, I. E. (2020). Mouse models of neural tube defects. *Adv. Exp. Med. Biol.* 1236, 39-64. 10.1007/978-981-15-2389-2_2 32304068

